# Bacterial diversity in Buruli ulcer skin lesions: Challenges in the clinical microbiome analysis of a skin disease

**DOI:** 10.1371/journal.pone.0181994

**Published:** 2017-07-27

**Authors:** Chloé Van Leuvenhaege, Koen Vandelannoote, Dissou Affolabi, Françoise Portaels, Ghislain Sopoh, Bouke C. de Jong, Miriam Eddyani, Conor J. Meehan

**Affiliations:** 1 Mycobacteriology unit, Department of Biomedical Sciences, Institute of Tropical Medicine, Antwerp, Belgium; 2 Laboratoire de Référence des Mycobactéries, Cotonou, Benin; 3 Institut Régionale de Santé Publique, CDTUB Allada, Benin; Institut de Pharmacologie et de Biologie Structurale, FRANCE

## Abstract

**Background:**

Buruli ulcer (BU) is an infectious disease caused by *Mycobacterium ulcerans* and considered the third most prevalent mycobacterial disease in humans. Secondary bacterial infections in open BU lesions are the main cause of pain, delayed healing and systemic illness, resulting in prolonged hospital stay. Thus, understanding the diversity of bacteria, termed the microbiome, in these open lesions is important for proper treatment. However, adequately studying the human microbiome in a clinical setting can prove difficult when investigating a neglected tropical skin disease due to its rarity and the setting.

**Methodology/Principal findings:**

Using 16S rRNA sequencing, we determined the microbial composition of 5 BU lesions, 3 non-BU lesions and 3 healthy skin samples. Although no significant differences in diversity were found between BU and non-BU lesions, the former were characterized by an increase of Bacteroidetes compared to the non-BU wounds and the BU lesions also contained significantly more obligate anaerobes. With this molecular-based study, we were also able to detect bacteria that were missed by culture-based methods in previous BU studies.

**Conclusions/Significance:**

Our study suggests that BU may lead to changes in the skin bacterial community within the lesions. However, in order to determine if such changes hold true across all BU cases and are either a cause or consequence of a specific wound environment, further microbiome studies are necessary. Such skin microbiome analysis requires large sample sizes and lesions from the same body site in many patients, both of which can be difficult for a rare disease. Our study proposes a pipeline for such studies and highlights several drawbacks that must be considered if microbiome analysis is to be utilized for neglected tropical diseases.

## Background

Buruli ulcer (BU) is an infectious disease caused by *Mycobacterium ulcerans* and considered the third most prevalent mycobacterial disease in humans. It is most often found in West and Central Africa, but it can also occur in Asia, Mexico and South America, the Western Pacific and Australia. [1,2] This skin disease can occur anywhere on the body, yet more commonly affects the extremities, with symptoms varying from nodules, papules, oedema to ulcers with undermined edges. [1,2] Experienced clinicians often diagnose BU based on clinical information only, initiating specific treatment while awaiting laboratory confirmation. [3] Knowing that every stage of BU development resembles clinical features of other diseases, especially on the lower limbs, this approach runs the risk that a wrong diagnosis is made, and inappropriate therapy prescribed. [4] This implies the importance of microbiological confirmation in the diagnosis of BU. However, even with antibiotic treatment available, secondary bacterial infections in open BU lesions are still prominent, causing pain, delayed healing and systemic illness, resulting in prolonged hospital stays, yet can be difficult to distinguish from colonization. [5–7] This warrants a closer look into the diversity of bacteria in these open lesions.

In healthy individuals, the skin forms a natural barrier against various toxic substances and pathogens. The totality of microorganisms and their collective genetic material present on the skin is called the skin microbiome. Under normal conditions, resident and transient bacteria within this microbiome will increase the resistance of the skin against pathogens through immunomodulation. However, some factors such as underlying diseases and ulceration may impair the immune defenses of the skin and create changed environments in which commensal bacteria may become opportunistic pathogens. These disrupted bacterial balances might impact the clinical outcome of the wounds. [8–11]

Characterization of bacteria in colonized and/or infected open BU lesions mostly happens in a clinical culture-dependent way. However, culture-based techniques select for the faster-growing bacteria, as some of these species flourish under certain laboratory conditions, while other species are out-competed, even when they can be cultured *in vivo*. [12] Until now the most prominent bacteria cultured from BU swabs were *Pseudomonas aeruginosa*, *Proteus mirabilis*, *Staphylococcus* sp., *Streptococcus* (Group A, B and C), and Enterobacteriaceae. [5,6] With molecular techniques such as 16S rRNA metagenomic sequencing, this bias is reduced, yielding a more precise overview of the bacteria present within the ulcers and their relative abundances.

The totality of microbes present on the human skin is referred to as the skin microbiome [11]. This bacterial diversity differs between body sites, primarily based on moisture levels [13]. Guidelines have been put forward for the clinical analysis of skin microbiome samples. Patients are to be excluded if antibiotics have been used in the past 7 days, there is cracking skin on the palms/soles, and many other criteria [14]. Additionally, since the microbiome differs between body sites, sufficient samples should be taken from the same site on different patients and controls for proper comparisons.

In this pilot study, we compared the microbial population associated with BU and non-BU skin samples from patients with ulcers of different etiology, collected in Benin, and healthy skin samples from similar body sites, collected from the same BU endemic area and from public datasets, to look for shifts in the microbial diversity associated with *M*. *ulcerans* disease. While the size of this study is small, it does highlight some of the pitfalls and difficulties associated with undertaking such tropical skin disease microbiome studies.

## Methods

### Sample overview

This microbiome study was nested within a study on *ex vivo* RNA expression and differential diagnosis of BU.[15] Ethical approval was obtained for the parent study from the ‘Comité National Provisoire d’Ethique pour la Recherche en Santé’ (CNPERS) in Benin (registration n°: IRB 00006860), the Institutional Review Board (IRB) of the Institute of Tropical Medicine (ITM), Antwerp (code: 11 25 4 778) and the Ethics committee of University Antwerp Hospital (UZA) (registration n°: B300201213080). Written informed consent was obtained from four patients. All other participants provided oral consent and did so after learning that surplus samples from their routine diagnostic or therapeutic procedure would be used to optimize molecular analyses on the bacteria causing their illness. Additionally, the healthy control was the mother of one of the study participants, who provided oral consent. For this add-on of retrospective de-identified analysis on these surplus samples on the microbiome of Buruli ulcer, no separate IRB approval has been sought. Patient data were anonymized for all laboratory and data analyses.

Samples were collected from nine patients for whom there was enough material leftover from the parent study at the Centre de Dépistage et de Traitement de l’Ulcère de Buruli (CDTUB) in Allada, Benin, in 2011. In that year, there had been a total of 80 patients clinically suspected of BU at the CDTUB. (Table 1) To be included in this study, patients could not have used antibiotics in the last month. All nine patients had open lesions and were clinically suspected of being infected with *M*. *ulcerans*. Five patients were confirmed BU (category 2: a single large ulcerative lesion 5–15 cm in diameter) by IS*2404* PCR, while the other four were IS*2404* PCR negative (non-BU) with skin lesions of unknown etiology. [16]

**Table 1:** Sample information.

Sample ID	Experimental group	Age	IS*2404* PCR	Sample type	Country of origin	Wound location	Antibiotics (Any kind)	Study	Lost after rarefaction
**BU1**	BU (Cat. 2)	20	+	Skin biopsy	Benin	Right leg	No	Pilot study	
**BU2**	BU (Cat. 2)	25	+	Skin biopsy	Benin	Right leg	No	Pilot study	
**BU3**	BU (Cat. 2)	18	+	Skin biopsy	Benin	Right leg	No	Pilot study	
**BU4**	BU (Cat. 2)	7	+	Skin biopsy	Benin	Left leg	No	Pilot study	
**BU5a**	BU (Cat. 2)	68	+	Skin biopsy	Benin	Buttock	No	Pilot study	
**BU5b**				Skin biopsy					
**Non-BU1a**	non-BU	33	-	Skin biopsy	Benin	Right arm	No	Pilot study	
**Non-BU1b**				Skin biopsy					X
**Non-BU2a**	non-BU	8	-	Skin biopsy	Benin	Left leg	No	Pilot study	X
**Non-BU2b**				Skin biopsy					X
**Non-BU3**	non-BU	40	-	Skin biopsy	Benin	Left leg	No	Pilot study	
**Non-BU4a**	non-BU	60	-	Skin biopsy	Benin	Left leg	No	Pilot study	X
**Non-BU4b**				Skin biopsy					
**H1**	Healthy			Skin swab	Benin	Volar forearm	No	Pilot study	
**H2**	Healthy			Skin swab	USA	Volar forearm	No	HMP*	
**H3**	Healthy			Skin swab	USA	Back of knee	No	Costello et al. (2009)	
**H4**	Healthy			Skin swab	The Netherlands	Upper buttock	No	Zeeuwen et al. (2012)	X
**Negative (GTC)**	Control							Pilot study	X
**Reagent Blank**	Control								X

* Human Microbiome Project

### Sample collection

The sampling was done after cleaning the lesions, using the following methods: purulent samples (skin biopsies) were taken from the necrotic tissues in the center of the lesion, from the necrotic tissues beneath the undermined edges of the ulcer, or punch biopsies (4 mm in diameter) under local anesthesia from surrounding tissues. In order to ensure the preservation of RNA (which is very unstable) for the parent study these biopsies were cut into pieces of 2–3 mm3 and added to an in-house non-sterile 5M Guanidium Thiocyanate (GTC) solution (13 ml) supplemented with 98 ml of 2-mercaptoethanol to stabilize the RNA within 5 minutes after taking the skin sample. After mixing the solution with the skin sample, the tube was stored for minimum 1 hour at room temperature after which the tube was transferred to a –80°C freezer. The samples were then shipped to the ITM (Antwerp, Blegium) on dry ice.

### DNA extraction

Total genomic DNA was extracted from the samples using the PowerSoil^TM^ DNA Isolation Kit (MO BIO Laboratories, Carlsbad, CA, US) according to the manufacturer’s protocol. After extraction, the DNA concentration was measured with the Qubit dsDNA BR assay kit (Invitrogen, Carlsbad, CA, USA) using the Qubit® 2.0 Fluorometer. The integrity of the gDNA was assessed by 1% agarose gel electrophoresis at 50 V for 2 hours. The extracted DNA was stored at -80°C before further PCR analysis.

### V3-V4 16S rRNA library preparation

A two-step, tailed PCR approach was used according to the Illumina protocol for 16S metagenomic sequencing library preparation. Both the V3 and V4 regions of the 16S ribosomal RNA genes were amplified with the S-D-Bact-0341-b-S-17 and S-D-Bact-0785-a-A-21 primers. The initial amplicon PCR reaction was conducted in a final volume of 25 μl (2x KAPA Hifi HotStart ReadyMix, 2.5 μl Microbial Genomic DNA (5 ng/μl in 10 mM Tris pH 8.5), and 10 μl of amplicon PCR primer mix (50/50 Forward/Reverse)). The cycling parameters used were: 3 min at 95°C, followed by 25 cycles of 30s at 95°C, 30s at 55°C and 30s at 72°C with a final extension at 72°C for 5 min after which the temperature was held at 4°C. Afterwards, the index PCR was performed with Nextera XT Index 1 Primers (N7XX) and Index 2 Primers (S5XX) from the Nextera XT Index Kit (Illumina), and with 5 μl amplicon derived from the previous PCR. The index PCR reaction was run in a final volume of 50 μl (2x KAPA Hifi HotStart ReadyMix, 5 μl DNA, 10 μl PCR Grade water, and 10 μl of Nextera XT Index Primer mix (50/50 primer1/primer2)). Limited cycling parameters were 3 min at 95°C, followed by 8 cycles of 30s at 95°C, 30s at 55°C and 30s at 72°C with a final extension at 72°C for 5 min and holding of the DNA at 4°C. The PCR products were both cleaned up using Agencourt AMPure XP beads (Beckman Coulter Genomics) and freshly prepared 80% ethanol. The average length and the integrity of the 16S amplicons were assessed by 1.5% agarose gel electrophoresis at 100 V for 30 min. The libraries were diluted to 4 nM using 10 mM Tris pH 8.5 and pooled together by aliquoting 5 μl of diluted DNA from each library and mixing them. Library denaturing, MiSeq sample loading and sequencing were done at the Centre of Medical Genetics of the University of Antwerp. In this run, also a minimum of 10% PhiX was included as an internal control for low diversity libraries. Paired-end sequencing was performed using MiSeq v3 reagents and 2x 300 bp reads.

### Selection of control samples

Several types of control samples were obtained for this study. One control sample was taken from the forearm of a healthy individual in Benin (a family member of one of the patients). The forearm was chosen because lesions on the upper limbs are more likely to be BU compared to lesions on the lower limbs. [17] The sample was stored and processed in the same way as our other clinical samples. For comparisons, we also included the data from three healthy individuals from three other publically available studies. Although attempts were made to find exact matches in terms of sequencing approaches, this was not possible. These control samples were chosen based on skin sites similar to our specimens and all were collected using swabs. 16S rRNA sequencing data from upper buttock (Dutch) and back of the knee (American) were obtained from the MG-RAST database of metagenomic studies [18] (project ids 2329 [19] and 81 [20]; 454-based V3-V4 and V2 sequencing respectively). Trimmed 16S metagenomic data (HM16STR) from an American volar forearm were obtained through the Human Microbiome Project Data Analysis and Coordination Center (HMP-DACC) [21] (study ID SRP002860; sample ID SRS144130; 454-based V3-V4 sequencing). Reads were obtained from the databases as post quality processed datasets but were further processed in the same manner as samples collected in this study. Additionally, to aid in identifying contamination, a negative control consisting only of the GTC buffer in which scissors and forceps were rinsed was collected in the same hospital, and also a reagent blank control consisting of all the reagents used during DNA extraction and 16S rRNA library preparation. The data set supporting the results of this article is available in the European Nucleotide Archive as project PRJEB14948, http://www.ebi.ac.uk/ena/data/view/PRJEB14948.

### Data analysis

Before processing Illumina sequencing data (See S1 Fig), we did an overall quality assessment of the raw reads using FastQC v.0.11.3. [22] The sequence read pairs were then combined using their overlapping portions, trimmed to remove very short (<45 bp) and very long (>610 bp) sequences and screened to remove reads with ambiguous nucleotide calls using the MOTHUR v.1.34.1 software [23] according to the standard operating procedure for the MiSeq system. [24] From these reads we removed the remaining human DNA by classifying them against a database of the human genome (GRcH38) with the Kraken tool (v. 0.10.5). [25] Chimeras were removed from our dataset in QIIME v.1.9.0 [26] by comparing them against the chimera-checked Greengenes 16S rRNA sequence database (v. 13.5) [27] using Usearch version 6.1. [28] Afterwards these reads were combined with the pre-processed reads from healthy swabs from the public studies. In order to remove other PCR artefacts and to keep reads of the right marker gene, we applied a prefiltering step at a cut off level of less than 60% similarity before picking operational taxonomic units (OTUs). Open-reference OTU picking was then performed consisting of a closed-reference OTU picking step that clusters the sequences to the Greengenes database at 97% similarity with UCLUST [29] and a *de novo* OTU picking step which clusters the unclassified sequences to each other at 97% similarity. [30] As a last step, the singletons were removed so that only OTUs with a minimum of two sequences were kept in the OTU table.

Next, we attempted to identify and remove potential contaminating OTUs deriving from reagents used during DNA extraction and 16S rRNA library preparation. For that purpose, we applied the method of Jervis-Bardy *et al*. [31] in which the relative abundances of the OTUs were correlated with the amplicon concentrations of the samples after library preparation in R v.3.0.0. [32] A significant inverse Spearman correlation would denote a contaminating OTU that needed to be filtered from our OTU table.

After contaminant removal, the OTU table was rarefied so that all samples were brought to a same sequencing depth of 1000 sequences. Second, since the 16S gene is present in multiple copies within some bacterial genomes, this variation in 16S copy number results in inflated counts for those species with high copy numbers. [33] We accounted for this bias by dividing the OTU counts by the predicted 16S copy number abundance of the associated species using PICRUSt (Online Galaxy version 1.0.0). [34] This generated a normalized OTU table with all OTUs still present, but with correct count numbers.

The α- and β-diversity measurements were undertaken using QIIME. Here we analyzed the bacterial diversity within the three groups (α-diversity) and compared the diversity between the three groups (β-diversity). For the α-diversity, we calculated the OTU richness using the Chao1 index, while the overall diversity (evenness) was measured with the Shannon Index and the Simpson’s Index. [35–37] For the β-diversity, we applied a non-phylogenetic based method, named the Bray-Curtis Index. [38] To measure the reliability of the estimates, we applied the jackknifing technique, in which we subsampled and calculated the Bray-Curtis index 100 times. These Bray-Curtis dissimilarities were used for a Principal Coordinates Analysis (PCoA).

To look for significant differences in α-diversity between BU, non-BU and healthy lesions a non-parametric two-sample t-test via Monte-Carlo permutation with Bonferroni multiple test correction (P≤0.05) was employed in QIIME. Statistical comparison of the metagenomes of the samples to distinguish ecological influences such as BU/non-BU disease was done using STAMP. [39] With this tool, taxonomic and compositional differences can be assessed between BU samples, non-BU samples and between the healthy microbiome and BU/non-BU microbiome, by looking at the abundance of metagenomic sequences. All two-way comparisons were done using the non-parametric Welch’s t-test with Benjamini-Hochberg FDR multiple test correction (P≤0.05). Reported p-values are those corrected for multiple testing.

## Results

From a total of 10,717,644 assembled reads, only 2,509,293 sequences were kept after quality filtering, pre-processing and removal of host-reads and chimeras. The high removal rate is due to the large amount of host DNA sequenced from the clinical samples. In order to ensure that all samples, both sequenced by this study and from public databases, met the same standards, a pre-filtering step was included. Although the reads of the samples from three healthy individuals (total: 2,577,253 sequences) were filtered in their respective studies, this pre-filtering step still resulted in a reduction of acceptable quality reads, resulting in a total of 2,332,978 sequences. This stringent filtering was used to ensure high confidence removal of any non-bacterial signal.

### OTU selection and taxonomy assignment

After clustering the sequences at a 97% similarity level with the open-reference OTU picking method and removing singletons, we observed a total of 7,903 different OTUs, from which 6,283 were picked *de novo* (i.e. not assigned to a specific known species). From these OTUs we also filtered potential contaminants, since these could influence the composition of our samples, especially those with a low bacterial content such as swabs (sample H1). A total of 61 OTUs, denoted by a significant inverse spearman correlation, were observed in the controls and samples, and filtered from the dataset since they were considered reagent-contaminating OTUs. (See S1 Table) Afterwards, another 161 OTUs were removed which were present in both the healthy swab H1 and the Blank reagent control. This lowered the overall number of OTUs to 7,681 (13–4,077 OTUs per sample). After rarefaction, only twelve samples remained in the dataset. Samples non-BU1b, Non-BU2 (a;b), Non-BU4a, and healthy control H4 were lost due to insufficient read numbers. (Table 1)

### Diversity analysis

**Fig 1:**
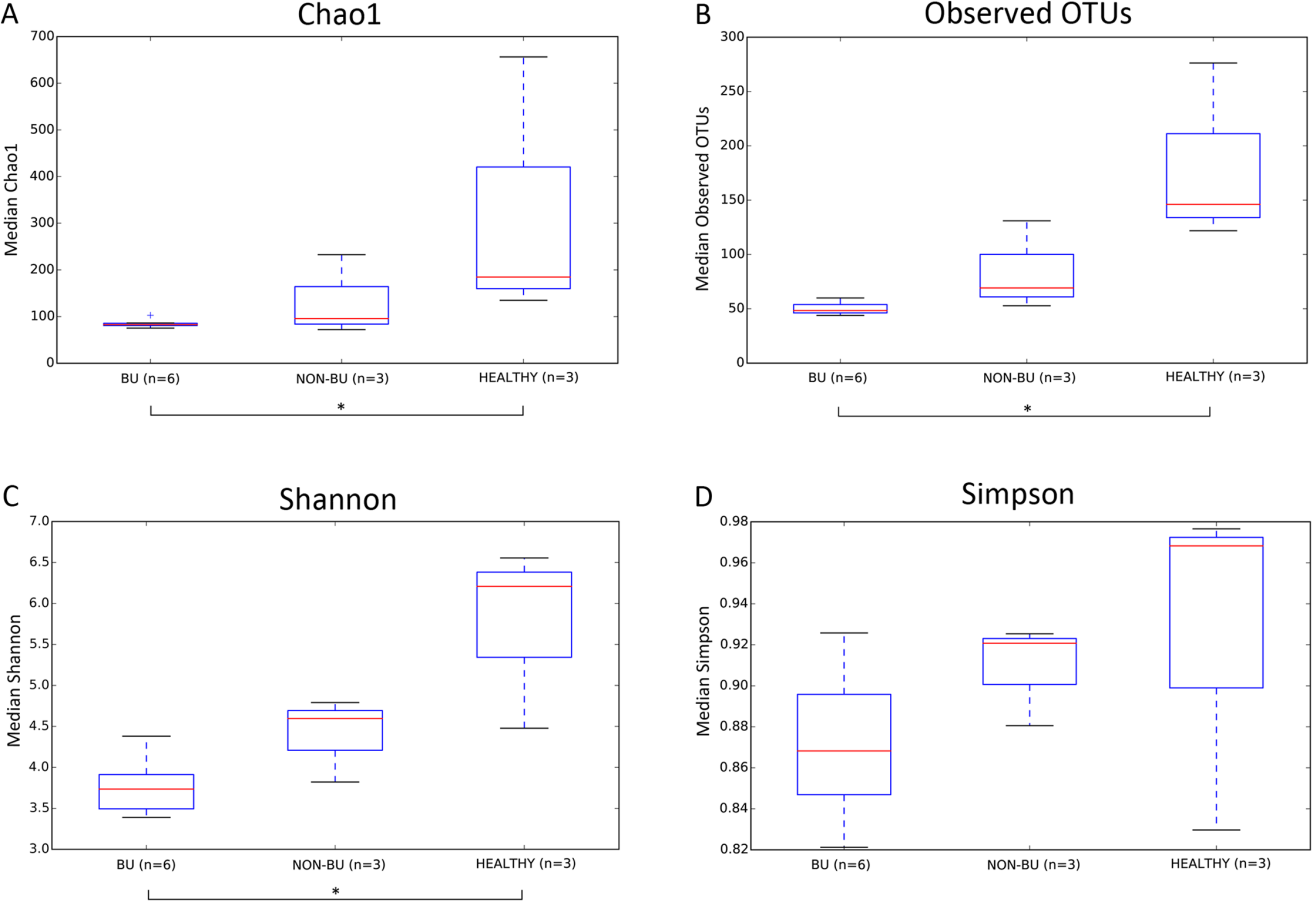
α-diversity calculations for BU (n = 6; 5 patients), non-BU (n = 3; 3 patients), and healthy (n = 3; 3 patients) samples. Whiskers in the boxplot represent minimum and maximum α-diversity values within the three groups. Significant differences were only seen between BU and healthy samples: (A) p = 0.027; (B) p = 0.009; (C) p = 0.036; non-parametric two sample t-test via Monte-Carlo permutation with Bonferroni multiple test correction.

Although skin microbiome samples are often only compared within body sites, the low sample size required comparison between all samples. The α-diversity calculations for all three groups are shown in Fig 1. Species richness (Fig 1A and 1B) was only significant between BU and healthy individuals (respectively p = 0.027 and p = 0.009), with a similar pattern observed in species evenness (p = 0.036). (Fig 1C) The overlapping outliers regarding the degree of dominance expressed by the Simpson index, showed no differences (Fig 1D).

**Fig 2:**
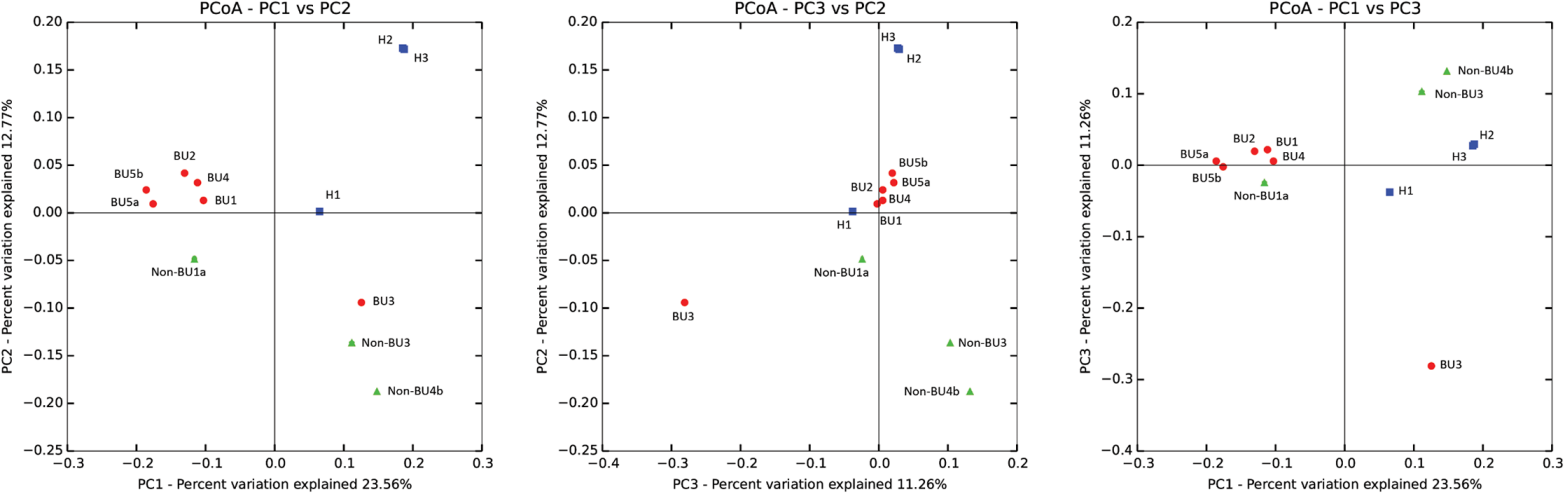
PCoA plot representing the distances between samples, expressed by the Bray-Curtis metric. Each point represents a different sample, while the colored circles, triangles, and squares represent BU, non-BU, and Healthy, respectively.

Differences in β-diversities were visualized using the Bray-Curtis distances between the three experimental groups in a PCoA plot. (Fig 2) In this plot the samples are projected based on differences regarding their OTUs and abundances. The first 2 principal coordinates (PC1 and PC2 representing respectively 23.56% and 12.77% of the total variance in the dataset) separate the three groups although sample H1 (Arm swab from a healthy resident in Benin) is separated from the other healthy samples (H2 and H3; swabs from US citizens), and shares more OTUs with the diseased groups. The BU samples group together, except for sample BU3, which resembles the non-BU samples more. This may be an indication for a BU related signal, although it is weak, especially in such a small dataset. If we remove the American samples (H2 and H3) so that only the Benin samples are compared, we see a separation with BU samples and non-BU1a on one side, and the healthy and other non-BU samples on the other side (PC1: 27.00% of total variance). However, there is still a big spread between healthy sample H1, BU sample BU3, and the two non-BU samples non-BU3 and non-BU4b. (S2 Fig)

### Taxonomic characterisation

**Fig 3:**
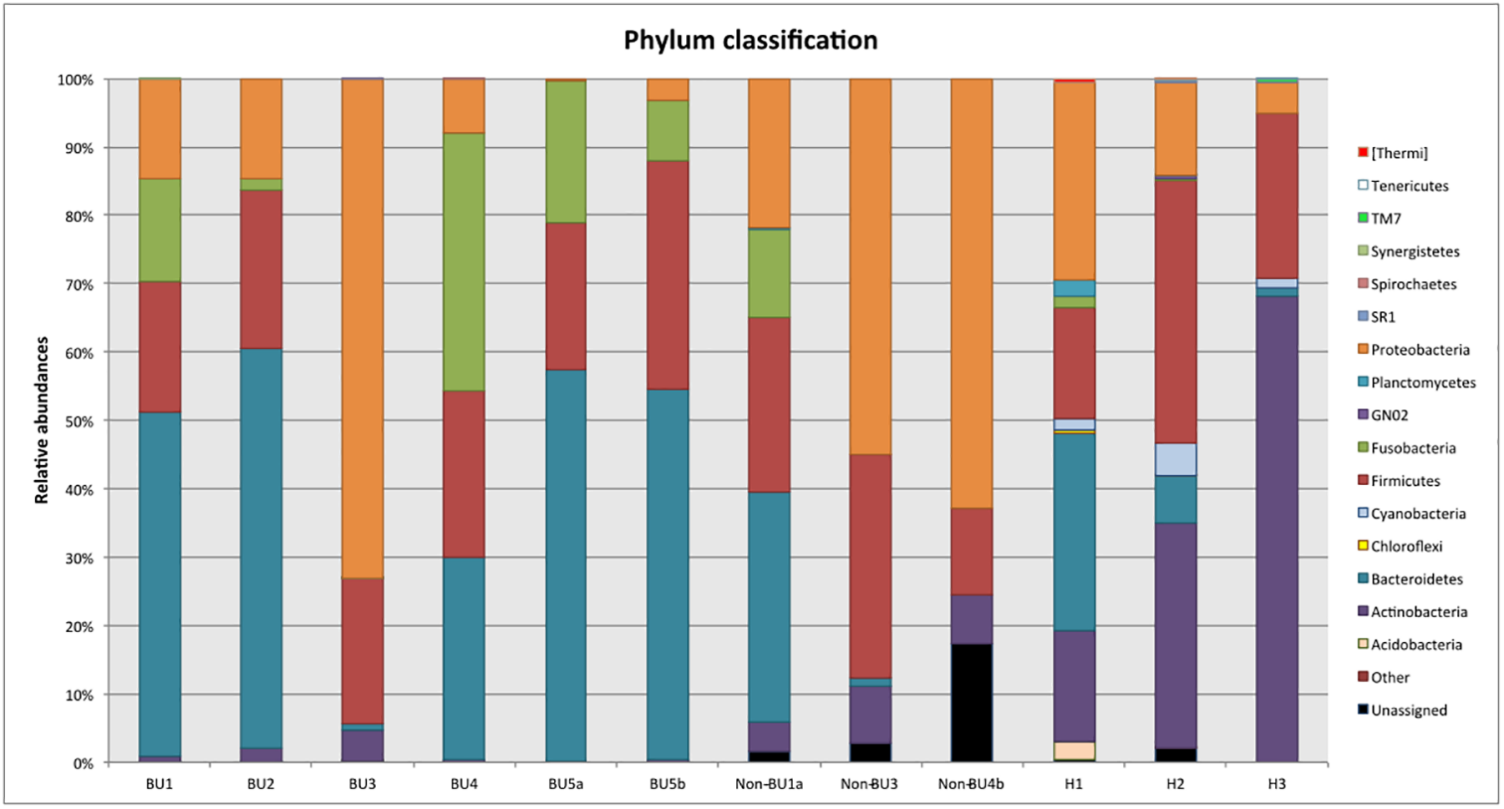
Phylum taxonomy level classifications. Bar plot showing the relative proportions of the phyla within all the samples.

Relative abundance comparisons can be a strong indicator of microbiome differences between disease conditions. Here, a comparison was done at the phylum level between samples. (Fig 3) The healthy skin samples, which act here as controls, were dominated by Actinobacteria (16.0–68.1%), as expected, but differed greatly in their phylum distributions based on country of origin. Comparisons between disease groups, however, showed no significant signals at the phylum level.

Overall, we identified 187 genera among which none showed significant differences in abundance between the three groups. (See S3 Fig) Among these 187, we detected the genus *Mycobacterium* in only three out of five BU patients (BU1; BU4; BU5a,b), despite all samples being IS*2404* PCR positive, but its relative abundance (0.0020%; 0.0147%; 0.0002% and 0.0001%, resp.) was only a small fraction of the total microbial composition.

### Gram-stain and oxygen tolerance

**Fig 4:**
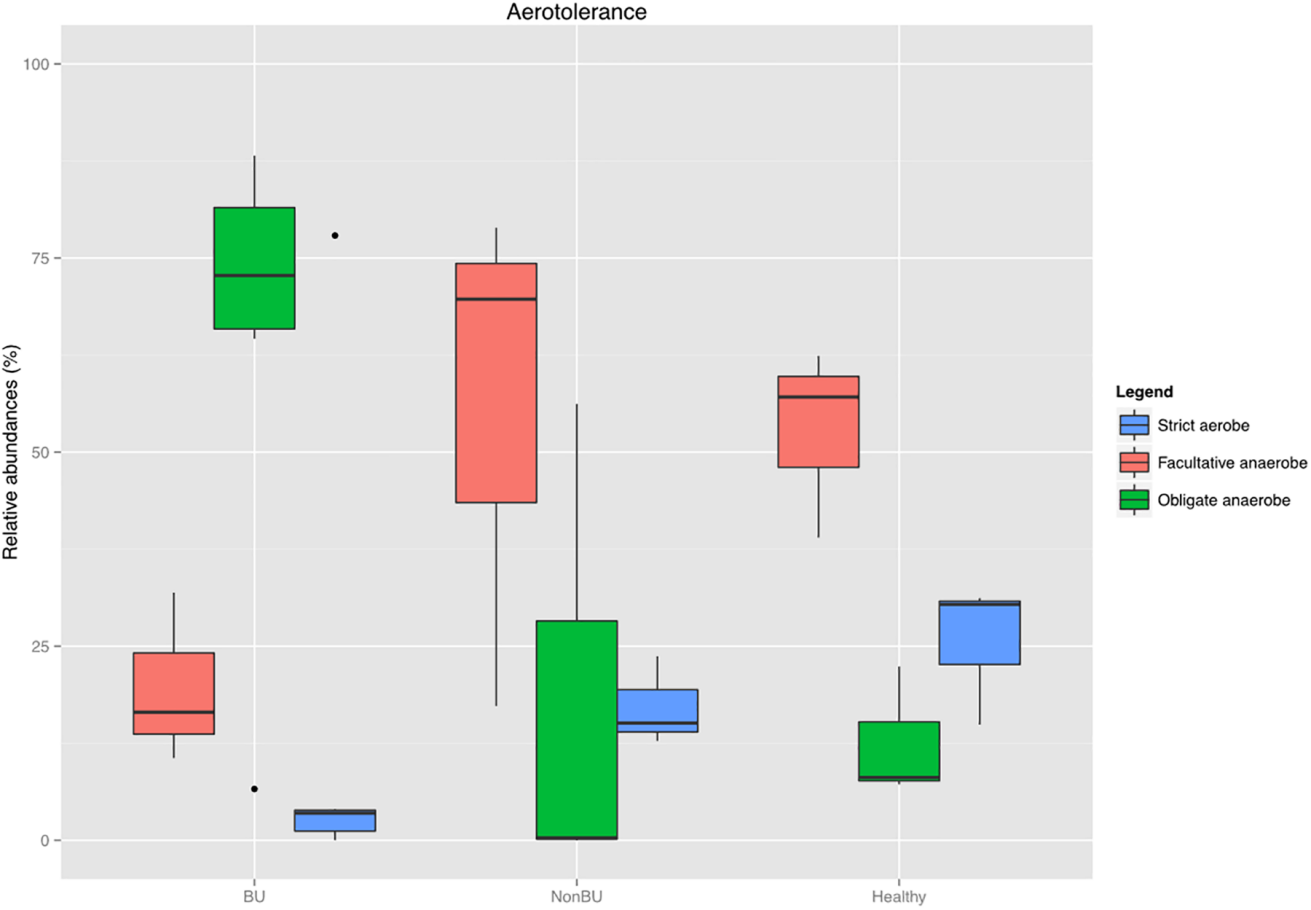
Oxygen tolerance within the three groups: Whiskers in the boxplot represent minimum and maximum values within the three groups. The BU lesions were almost entirely dominated by obligate anaerobes, which was significantly different from the healthy samples (p = 0.0325; Welch’s t-test with Benjamini-Hochberg FDR multiple test correction). The Non-BU group showed an increase in facultative anaerobic bacteria.

The physical properties of the bacterial cell wall (Gram-positive or -negative) and the oxygen tolerance of the bacteria are also very important factors in infection. Thus, we compared the relative abundances of Gram-positive and Gram-negative microbes, and their aerotolerance across the three groups. No significant difference was seen in terms of gram-positive versus gram-negative bacteria (S4 Fig). When comparing the oxygen tolerance, we observed a high abundance of strictly aerobe bacteria for the healthy skin (25.50 ± 7.50%), as was expected. (Fig 4) The BU lesions were almost entirely dominated by obligate anaerobes (64.72 ± 27.16%), which was significantly different from the healthy samples (p = 0.0325), but not from the non-BU samples (p = 0.56). This is in sharp contrast with non-BU lesions, which contained a high proportion of facultative anaerobes (55.30 ± 27.13%).

## Discussion

Using 16S rRNA sequencing we were able to define the microbial composition of several BU and non-BU lesions. Although weak signals of differences were observed, our low sample size did not allow us to draw definite conclusions. However, the investigation of this sample set highlights many pitfalls and measurements to be accounted for when undertaking clinical microbiome studies.

### Microbiome studies may uncover previously unassociated bacteria

Although few samples were processed, an interesting finding is that most of the uncovered strictly anaerobe bacteria were never cultured in previous BU studies, stressing the importance of whole community studies like the one presented here. [5–7] Such differences between culture and metagenomics sampling have been seen previously [40], suggesting that microbiome analyses should complement culture-based studies to uncover the full complement of bacterial diversity. What also could have also led to the lack of detection of strictly anaerobe bacteria in previous BU studies is the application of a different sampling method. By using superficial swabs it is possible that these anaerobes could not be detected, while skin biopsies go deeper into the ulcers thereby easily reaching anaerobic regions. Another explanation for their absence is the lack of effort and time in culturing these obligate anaerobes. After a short incubation period only those bacteria that grow easily on culture media are detected. [41,42]

### Setting-specific aspects of microbiome studies in resource-limited countries

This study was undertaken as a pilot investigation into the feasibility of a skin ulcer microbiome analysis in a tropical setting. Many limitations and obstacles were presented during the course of this study. Firstly, several problems are introduced due to the setting of the disease. Microbiome sequencing protocols will result in data derived from any and all microbes, including contaminants, as seen in previous clinical microbiome studies [40]. Therefore, it is important to ensure that samples are kept contamination-free as much as possible. Obtaining sterile material such as storage buffer, swabs and transport equipment can be difficult in remote African health centers where most BU cases are observed, especially when transport and storage is required for sequencing. Reagent contamination can be mitigated with statistical methods as outlined by Jervis-Bardy *et al*. [31] but other contaminations may still influence results in complex community analyses. Another important step in ensuring the correct community is sampled is refraining from wound cleaning and antibiotic treatment prior to sampling. These are essential steps to avoid perturbation of the microbial communities inside the ulcers. [11] These are also unlikely occurrences in such settings where antibiotic use is often undertaken without proper regimens or prescriptions [43]. Improvements could also be made through application of one sampling method (skin biopsies) on a fixed location (or multiple locations) within the ulcers in order to control for the microhabitats within the wound, each of which is characterized by their physiological parameters and bacterial composition. [44,45] Finding which area is most relevant for sampling is difficult, but warrants investigation. However, comparisons between healthy samples (likely swabs) and lesions (routinely biopsies) may be difficult, especially as even some biopsies did not contain adequate bacterial DNA for use within this study (as can be seen with exclusion at the rarefaction stage). This may be due to sample fixation at the site or biological reasons such as sparse bacterial communities within lesions. Sampling differences must also be taken into account when attempting to use controls derived from public data. Such data should be comparable (extraction protocol, 16S region sequenced etc.) to the undertaken study to ensure all biases are removed. Thus, we suggest that the standard human microbiome project (HMP) sequencing protocols should be followed for comparability, as was done in this study.

### Appropriate controls are crucial for uncovering BU-specific signals

For future BU microbiome studies to increase chances of finding significant differences not only do we want better quality samples and an increased sample size, but also appropriate controls. The age range should be similar for the three groups (BU, non-BU and healthy individuals, as was done here). In general, BU patients are younger than non-BU patients with children between 5–15 years mostly affected. [2,46,47] Since many of the bacteria detected in our BU and non-BU samples are members of the gut flora, it is important to note that such compositions can change with age [48][49] The age of the patients may thus play a role. In addition to ensuring a wide, yet comparable, spread of ages, samples from contralateral unaffected skin are needed to control for genetic differences, for specific body sites with diverse ecology and bacterial distribution, and to compare with as healthy skin controls. [8,20] Such samples are often not obtained routinely, making retrospective microbiome studies often infeasible and therefore requiring dedicated prospective studies to be undertaken. Furthermore, more control samples are needed from healthy individuals from the same geographical area rather than from public databases, as the microbiome of these patients may be influenced by confounders such as ethnicity, geography, daily activities, etc. [50] This was observed here as the public database American samples differed greatly from the Benin healthy control. A larger database of African-derived microbiome samples is needed if correct controls are to be provided for such studies. Also the usage of negative controls and blank reagent controls is important to determine potential contaminants derived from the storage buffer, and reagents and kits used during sample processing, especially for clinical samples such as these. [51] Lastly, it is important to keep in mind that microbiome studies cannot always determine the actual cause of an infection by comparing the prevalence and abundances of pathogens, as evidenced by the low abundance of the known causative agent, *M*. *ulcerans*. [52] Only three of five PCR-confirmed BU samples had reads mapped to the *Mycobacterium* genus. This may be due to low DNA yield, insufficient high-quality reads or overly stringent filtering steps. However, several studies of TB-associated microbiomes also found little or no *M*. *tuberculosis*-associated reads in positive samples. [53–55] This suggests that the causative agent of the disease may not be highly abundant in the sample and that such sequencing methods may not be appropriate for clinical detection of mycobacteria.

### Small samples sizes must be overcome with large prospective studies

Lastly, as with many neglected tropical diseases, an adequate sample size is difficult to obtain. Statistical estimation of a sample size was not possible since prior information on BU microbiome studies is non-existent. Therefore, it requires estimating the correct sample size based on studies with a ‘similar’ setup. Studies comparing healthy skin and psoriatic lesions, with skin biopsies as a sampling method had 10–15 patients per sample group [56,57]. As samples should ideally be compared within body site, this requires 30 samples per body site (10 each from BU, non-BU and a healthy control group). This is likely difficult for neglected diseases that are not restricted to a specific body site such as BU, where cases in the whole world were only 2037 in 32015 [58], and would require local non-BU diseases with exact body site matching for proper comparisons. This is not a problem restricted to BU, as similar numbers would be required for other neglected tropical skin diseases such as yaws (up to 20,000 cases per year in some countries [59]) and even more prominent skin diseases such as cutaneous leishmaniasis (700,00–1.2 million cases per year [60]) and leprosy (250,000 cases per year [61]), for which a previous microbiome study was undertaken but with no matching non-leprosy lesions or local healthy controls [62]. However, although difficult, it is not infeasible as 80 clinical BU suspects were seen in the CDTUB in 2011 and the parent study recruited 135 such patients.

### Recommendations for future BU-associated microbiome studies

This pilot study demonstrates some potential trends of bacterial diversity differences between BU, non-BU and healthy skin environments. In order to determine if these alterations were due to a specific wound environment, underlying pathophysiological conditions created by *M*. *ulcerans*, or wound location on the body, and if there is a specific microbial shift associated with BU lesions, further microbiome studies with a larger sample size, standardized methodology and appropriate study design are necessary Such a study would need to be prospective, to ensure intra-patient controls are taken, and to match healthy and non-BU samples to body sites. It would also need to last long enough to reach sufficient numbers of samples. An optimal sampling approach (swab or biopsy) comparable with healthy controls is required and perhaps with samples within closely matching public databases, especially in terms of geographic origin. Patients should be excluded based on certain criteria such as antibiotic usage and any DNA extraction and sequencing should follow the recommended HMP protocol, as was used here. If such stipulations are followed, the data analysis pipeline outlined within this study would provide ample power and resolution to uncover any changes in the microbiome associated with BU lesions.

## Supporting information

S1 Fig Workflow of data analysis and statistics. (PDF)

S2 Fig PCoA plot representing the distances between samples, expressed by the Bray-Curtis metric. Each point represents a different sample, while the colored circles, triangles, and squares represent BU, non-BU, and Healthy, respectively. In this plot we did not integrate the two samples from the healthy US citizens.
(PNG)

S3 Fig Taxa summary at genus-level. (PDF)

S4 Fig Distribution of Gram-positivity and -negativity among the experimental groups. Whiskers in the boxplot represent minimum and maximum values within the three groups. The results show an increase in Gram-negative bacteria within the diseased groups compared to the healthy individuals, although this is not significant.
(TIFF)

S1 Table Contaminating OTUs. This table contains the OTUs and the genera and families (*) they represent. These OTUs were considered contaminants due to a significant inverse spearman correlation and were removed from the dataset.
(DOCX)

## References

[1] van der Werf TS, Stienstra Y, Johnson RC, Phillips R, Adjei O, Fleischer B, et al Mycobacterium ulcerans disease. Bull World Health Organ. World Health Organization; 2005;83: 785–91. 16283056 PMC2626418

[2] Walsh DS, Portaels F, Meyers WM. Buruli ulcer: Advances in understanding Mycobacterium ulcerans infection. Dermatol Clin. 2011;29: 1–8. doi: 10.1016/j.det.2010.09.006 21095521 10.1016/j.det.2010.09.006

[3] Organisation mondiale de la Santé. Traitement de l’infection à mycobacterium ulcerans (ulcère de Buruli): recommandations à l’intention des agents de santé. Organisation mondiale de la Santé; 2012;

[4] Kibadi K, Boelaert M, Fraga AG, Kayinua M, Longatto-Filho A, Minuku J-B, et al Response to treatment in a prospective cohort of patients with large ulcerated lesions suspected to be Buruli Ulcer (Mycobacterium ulcerans disease). PLoS Negl Trop Dis. 2010;4: e736 doi: 10.1371/journal.pntd.0000736 20625556 10.1371/journal.pntd.0000736 PMC2897843

[5] Barogui YT, Klis S, Bankolé HS, Sopoh GE, Mamo S, Baba-Moussa L, et al Towards Rational Use of Antibiotics for Suspected Secondary Infections in Buruli Ulcer Patients. Small PLC, editor. PLoS Negl Trop Dis. Public Library of Science; 2013;7: e2010 doi: 10.1371/journal.pntd.0002010 23359827 10.1371/journal.pntd.0002010 PMC3554522

[6] Yeboah-Manu D, Kpeli GS, Ruf M-T, Asan-Ampah K, Quenin-Fosu K, Owusu-Mireku E, et al Secondary bacterial infections of buruli ulcer lesions before and after chemotherapy with streptomycin and rifampicin. PLoS Negl Trop Dis. 2013;7: e2191 doi: 10.1371/journal.pntd.0002191 23658847 10.1371/journal.pntd.0002191 PMC3642065

[7] Amissah NA, Glasner C, Ablordey A, Tetteh CS, Kotey NK, Prah I, et al Genetic diversity of Staphylococcus aureus in Buruli ulcer. PLoS Negl Trop Dis. 2015;9: e0003421 doi: 10.1371/journal.pntd.0003421 25658641 10.1371/journal.pntd.0003421 PMC4319846

[8] Grice EA, Kong HH, Conlan S, Deming CB, Davis J, Young AC, et al Topographical and temporal diversity of the human skin microbiome. Science. NIH Public Access; 2009;324: 1190–2. doi: 10.1126/science.1171700 19478181 10.1126/science.1171700 PMC2805064

[9] Grice EA, Kong HH, Renaud G, Young AC, NISC Comparative Sequencing Program, Bouffard GG, et al A diversity profile of the human skin microbiota. Genome Res. 2008;18: 1043–50. doi: 10.1101/gr.075549.107 18502944 10.1101/gr.075549.107 PMC2493393

[10] Grice EA, Segre JA. Interaction of the microbiome with the innate immune response in chronic wounds. Adv Exp Med Biol. 2012;946: 55–68. doi: 10.1007/978-1-4614-0106-3_4 21948362 10.1007/978-1-4614-0106-3_4 PMC3516280

[11] Grice EA, Segre JA. The skin microbiome. Nat Rev Microbiol. Nature Publishing Group; 2011;9: 244–253. doi: 10.1038/nrmicro2537 21407241 10.1038/nrmicro2537 PMC3535073

[12] Rhoads DD, Cox SB, Rees EJ, Sun Y, Wolcott RD. Clinical identification of bacteria in human chronic wound infections: culturing vs. 16S ribosomal DNA sequencing. BMC Infect Dis. 2012;12: 321 doi: 10.1186/1471-2334-12-321 23176603 10.1186/1471-2334-12-321 PMC3542000

[13] Tagami H. Location-related differences in structure and function of the stratum corneum with special emphasis on those of the facial skin. Int J Cosmet Sci. 2008;30: 413–34. doi: 10.1111/j.1468-2494.2008.00459.x 19099543 10.1111/j.1468-2494.2008.00459.x

[14] McInnes P, Cutting M. Manual of Procedures for Human Microbiome Project [Internet]. 2010. Available: http://www.hmpdacc.org/doc/HMP_MOP_Version12_0_072910.pdf

[15] Eddyani M, Affolabi D, Brun L, Roux JJ, Ayelo G, Yves Barogui DA, et al. The accuracy of the clinical and microbiological diagnosis of Buruli ulcer. WHO meeting on Buruli Ulcer. Geneva; 2015.

[16] Stinear T, Ross BC, Davies JK, Marino L, Robins-Browne RM, Oppedisano F, et al Identification and characterization of IS2404 and IS2606: two distinct repeated sequences for detection of Mycobacterium ulcerans by PCR. J Clin Microbiol. 1999;37: 1018–23. Available: http://www.ncbi.nlm.nih.gov/pubmed/10074520 10074520 10.1128/jcm.37.4.1018-1023.1999 PMC88643

[17] Mueller YK, Bastard M, Nkemenang P, Comte E, Ehounou G, Eyangoh S, et al The “Buruli Score”: Development of a Multivariable Prediction Model for Diagnosis of Mycobacterium ulcerans Infection in Individuals with Ulcerative Skin Lesions, Akonolinga, Cameroon. PLoS Negl Trop Dis. Public Library of Science; 2016;10: e0004593 doi: 10.1371/journal.pntd.0004593 27045293 10.1371/journal.pntd.0004593 PMC4821558

[18] Meyer F, Paarmann D, D’Souza M, Olson R, Glass EM, Kubal M, et al The metagenomics RAST server—a public resource for the automatic phylogenetic and functional analysis of metagenomes. BMC Bioinformatics. 2008;9: 386 doi: 10.1186/1471-2105-9-386 18803844 10.1186/1471-2105-9-386 PMC2563014

[19] Zeeuwen PLJM, Boekhorst J, van den Bogaard EH, de Koning HD, van de Kerkhof PMC, Saulnier DM, et al Microbiome dynamics of human epidermis following skin barrier disruption. Genome Biol. 2012;13: R101 doi: 10.1186/gb-2012-13-11-r101 23153041 10.1186/gb-2012-13-11-r101 PMC3580493

[20] Costello EK, Lauber CL, Hamady M, Fierer N, Gordon JI, Knight R. Bacterial community variation in human body habitats across space and time. Science (80-). 2009;326: 1694–7. doi: 10.1126/science.1177486 19892944 10.1126/science.1177486 PMC3602444

[21] Wortman J, Giglio M, Creasy H, Chen A, Liolios K, Chu K, et al A data analysis and coordination center for the human microbiome project. Genome Biol. BioMed Central; 2010;11: O13 doi: 10.1186/gb-2010-11-s1-o13

[22] Andrews S. FastQC A Quality Control tool for High Throughput Sequence Data [Internet]. 2010. Available: http://www.bioinformatics.babraham.ac.uk/projects/fastqc

[23] Schloss PD, Westcott SL, Ryabin T, Hall JR, Hartmann M, Hollister EB, et al Introducing mothur: open-source, platform-independent, community-supported software for describing and comparing microbial communities. Appl Environ Microbiol. 2009;75: 7537–41. doi: 10.1128/AEM.01541-09 19801464 10.1128/AEM.01541-09 PMC2786419

[24] Kozich JJ, Westcott SL, Baxter NT, Highlander SK, Schloss PD. Development of a dual-index sequencing strategy and curation pipeline for analyzing amplicon sequence data on the MiSeq Illumina sequencing platform. Appl Environ Microbiol. American Society for Microbiology; 2013;79: 5112–20. doi: 10.1128/AEM.01043-13 23793624 10.1128/AEM.01043-13 PMC3753973

[25] Wood DE, Salzberg SL, Venter C, Remington K, Heidelberg J, Halpern A, et al Kraken: ultrafast metagenomic sequence classification using exact alignments. Genome Biol. BioMed Central; 2014;15: R46 doi: 10.1186/gb-2014-15-3-r46 24580807 10.1186/gb-2014-15-3-r46 PMC4053813

[26] Caporaso JG, Kuczynski J, Stombaugh J, Bittinger K, Bushman FD, Costello EK, et al QIIME allows analysis of high-throughput community sequencing data. Nat Methods. Nature Publishing Group; 2010;7: 335–336. doi: 10.1038/nmeth.f.303 20383131 10.1038/nmeth.f.303 PMC3156573

[27] DeSantis TZ, Hugenholtz P, Larsen N, Rojas M, Brodie EL, Keller K, et al Greengenes, a chimera-checked 16S rRNA gene database and workbench compatible with ARB. Appl Environ Microbiol. 2006;72: 5069–72. doi: 10.1128/AEM.03006-05 16820507 10.1128/AEM.03006-05 PMC1489311

[28] Edgar RC, Haas BJ, Clemente JC, Quince C, Knight R. UCHIME improves sensitivity and speed of chimera detection. Bioinformatics. 2011;27: 2194–200. doi: 10.1093/bioinformatics/btr381 21700674 10.1093/bioinformatics/btr381 PMC3150044

[29] Edgar RC. Search and clustering orders of magnitude faster than BLAST. Bioinformatics. 2010;26: 2460–1. doi: 10.1093/bioinformatics/btq461 20709691 10.1093/bioinformatics/btq461

[30] Rideout JR, He Y, Navas-Molina JA, Walters WA, Ursell LK, Gibbons SM, et al Subsampled open-reference clustering creates consistent, comprehensive OTU definitions and scales to billions of sequences. PeerJ. 2014;2: e545 doi: 10.7717/peerj.545 25177538 10.7717/peerj.545 PMC4145071

[31] Jervis-Bardy J, Leong LEX, Marri S, Smith RJ, Choo JM, Smith-Vaughan HC, et al Deriving accurate microbiota profiles from human samples with low bacterial content through post-sequencing processing of Illumina MiSeq data. Microbiome. BioMed Central; 2015;3: 19 doi: 10.1186/s40168-015-0083-8 25969736 10.1186/s40168-015-0083-8 PMC4428251

[32] Team RC. R: A language and environment for statistical computing. [Internet]. R Foundation for Statistical Computing, Vienna, Austria; 2014. Available: http://www.r-project.org/

[33] Kembel SW, Wu M, Eisen JA, Green JL, Hebert P, Cywinska A, et al Incorporating 16S Gene Copy Number Information Improves Estimates of Microbial Diversity and Abundance. von Mering C, editor. PLoS Comput Biol. Public Library of Science; 2012;8: e1002743 doi: 10.1371/journal.pcbi.1002743 23133348 10.1371/journal.pcbi.1002743 PMC3486904

[34] Langille MGI, Zaneveld J, Caporaso JG, McDonald D, Knights D, Reyes JA, et al Predictive functional profiling of microbial communities using 16S rRNA marker gene sequences. Nat Biotechnol. Nature Research; 2013;31: 814–821. doi: 10.1038/nbt.2676 23975157 10.1038/nbt.2676 PMC3819121

[35] Chao A. Nonparametric Estimation of the Number of Classes in a Population. Scand J Stat. [Board of the Foundation of the Scandinavian Journal of Statistics, Wiley]; 1984;11: 265–270.

[36] Shannon CE. A Mathematical Theory of Communication. Bell Syst Tech J. Blackwell Publishing Ltd; 1948;27: 379–423. doi: 10.1002/j.1538-7305.1948.tb01338.x

[37] SIMPSON EH, E. H. Measurement of Diversity. Nature. 1949;163: 688–688. doi: 10.1038/163688a0

[38] Bray JR, Curtis JT. An Ordination of the Upland Forest Communities of Southern Wisconsin. Ecol Monogr. 1957;27: 325–349. doi: 10.2307/1942268

[39] Parks DH, Beiko RG. Identifying biologically relevant differences between metagenomic communities. Bioinformatics. 2010;26: 715–21. doi: 10.1093/bioinformatics/btq041 20130030 10.1093/bioinformatics/btq041

[40] Decuypere S, Meehan CJ, Van Puyvelde S, De Block T, Maltha J, Palpouguini L, et al Diagnosis of Bacterial Bloodstream Infections: A 16S Metagenomics Approach. PLoS Negl Trop Dis. 2016;10: e0004470 doi: 10.1371/journal.pntd.0004470 26927306 10.1371/journal.pntd.0004470 PMC4771206

[41] Bowler PG, Duerden BI, Armstrong DG. Wound microbiology and associated approaches to wound management. Clin Microbiol Rev. American Society for Microbiology (ASM); 2001;14: 244–69. doi: 10.1128/CMR.14.2.244-269.2001 11292638 10.1128/CMR.14.2.244-269.2001 PMC88973

[42] Davies CE, Wilson MJ, Hill KE, Stephens P, Hill CM, Harding KG, et al Use of molecular techniques to study microbial diversity in the skin: chronic wounds reevaluated. Wound Repair Regen. 9: 332–40. Available: http://www.ncbi.nlm.nih.gov/pubmed/11896975 11896975 10.1046/j.1524-475x.2001.00332.x

[43] Morgan DJ, Okeke IN, Laxminarayan R, Perencevich EN, Weisenberg S. Non-prescription antimicrobial use worldwide: a systematic review. Lancet Infect Dis. NIH Public Access; 2011;11: 692–701. doi: 10.1016/S1473-3099(11)70054-8 21659004 10.1016/S1473-3099(11)70054-8 PMC3543997

[44] Kirkup BC. Bacterial Strain Diversity Within Wounds. Adv wound care. 2015;4: 12–23. doi: 10.1089/wound.2014.0560 25566411 10.1089/wound.2014.0560 PMC4281850

[45] Price LB, Liu CM, Frankel YM, Melendez JH, Aziz M, Buchhagen J, et al Macroscale spatial variation in chronic wound microbiota: a cross-sectional study. Wound Repair Regen. 19: 80–8. doi: 10.1111/j.1524-475X.2010.00628.x 20946140 10.1111/j.1524-475X.2010.00628.x PMC3022109

[46] Merritt RW, Walker ED, Small PLC, Wallace JR, Johnson PDR, Benbow ME, et al Ecology and transmission of Buruli ulcer disease: a systematic review. PLoS Negl Trop Dis. 2010;4: e911 doi: 10.1371/journal.pntd.0000911 21179505 10.1371/journal.pntd.0000911 PMC3001905

[47] Huang GKL, Johnson PDR. Epidemiology and management of Buruli ulcer. Expert Rev Anti Infect Ther. 2014;12: 855–65. doi: 10.1586/14787210.2014.910113 24918117 10.1586/14787210.2014.910113

[48] Mariat D, Firmesse O, Levenez F, Guimarăes V, Sokol H, Doré J, et al The Firmicutes/Bacteroidetes ratio of the human microbiota changes with age. BMC Microbiol. BioMed Central; 2009;9: 123 doi: 10.1186/1471-2180-9-123 19508720 10.1186/1471-2180-9-123 PMC2702274

[49] Langille MG, Meehan CJ, Koenig JE, Dhanani AS, Rose RA, Howlett SE, et al Microbial shifts in the aging mouse gut. Microbiome. 2014;2: 50 doi: 10.1186/s40168-014-0050-9 25520805 10.1186/s40168-014-0050-9 PMC4269096

[50] Hospodsky D, Pickering AJ, Julian TR, Miller D, Gorthala S, Boehm AB, et al Hand bacterial communities vary across two different human populations. Microbiology. 2014;160: 1144–52. doi: 10.1099/mic.0.075390-0 24817404 10.1099/mic.0.075390-0

[51] Salter SJ, Cox MJ, Turek EM, Calus ST, Cookson WO, Moffatt MF, et al Reagent and laboratory contamination can critically impact sequence-based microbiome analyses. BMC Biol. BioMed Central; 2014;12: 87 doi: 10.1186/s12915-014-0087-z 25387460 10.1186/s12915-014-0087-z PMC4228153

[52] Crisp JG, Takhar SS, Moran GJ, Krishnadasan A, Dowd SE, Finegold SM, et al Inability of polymerase chain reaction, pyrosequencing, and culture of infected and uninfected site skin biopsy specimens to identify the cause of cellulitis. Clin Infect Dis. 2015;61: 1679–87. doi: 10.1093/cid/civ655 26240200 10.1093/cid/civ655

[53] Wu J, Liu W, He L, Huang F, Chen J, Cui P, et al Sputum microbiota associated with new, recurrent and treatment failure tuberculosis. Neyrolles O, editor. PLoS One. Public Library of Science; 2013;8: e83445 doi: 10.1371/journal.pone.0083445 24349510 10.1371/journal.pone.0083445 PMC3862690

[54] Zhou Y, Lin F, Cui Z, Zhang X, Hu C, Shen T, et al Correlation between Either Cupriavidus or Porphyromonas and Primary Pulmonary Tuberculosis Found by Analysing the Microbiota in Patients’ Bronchoalveolar Lavage Fluid. Cardona P-J, editor. PLoS One. Public Library of Science; 2015;10: e0124194 doi: 10.1371/journal.pone.0124194 26000957 10.1371/journal.pone.0124194 PMC4441454

[55] Cheung MK, Lam WY, Fung WYW, Law PTW, Au CH, Nong W, et al Sputum microbiota in tuberculosis as revealed by 16S rRNA pyrosequencing. Bereswill S, editor. PLoS One. Public Library of Science; 2013;8: e54574 doi: 10.1371/journal.pone.0054574 23365674 10.1371/journal.pone.0054574 PMC3554703

[56] Gao Z, Tseng C, Strober BE, Pei Z, Blaser MJ. Substantial alterations of the cutaneous bacterial biota in psoriatic lesions. Ahmed N, editor. PLoS One. Public Library of Science; 2008;3: e2719 doi: 10.1371/journal.pone.0002719 18648509 10.1371/journal.pone.0002719 PMC2447873

[57] Fahlén A, Engstrand L, Baker BS, Powles A, Fry L. Comparison of bacterial microbiota in skin biopsies from normal and psoriatic skin. Arch Dermatol Res. Springer-Verlag; 2012;304: 15–22. doi: 10.1007/s00403-011-1189-x 22065152 10.1007/s00403-011-1189-x

[58] Organisation WH. Buruli Ulcer fact sheet [Internet]. 2016. Available: http://www.who.int/mediacentre/factsheets/fs199/en/

[59] Kazadi WM, Asiedu KB, Agana N, Mitjà O. Epidemiology of yaws: an update. Clin Epidemiol. Dove Press; 2014;6: 119–28. doi: 10.2147/CLEP.S44553 24729728 10.2147/CLEP.S44553 PMC3979691

[60] Alvar J, Vélez ID, Bern C, Herrero M, Desjeux P, Cano J, et al Leishmaniasis worldwide and global estimates of its incidence. PLoS One. Public Library of Science; 2012;7: e35671 doi: 10.1371/journal.pone.0035671 22693548 10.1371/journal.pone.0035671 PMC3365071

[61] Rodrigues LC, Lockwood DN. Leprosy now: epidemiology, progress, challenges, and research gaps. Lancet Infect Dis. 2011;11: 464–70. doi: 10.1016/S1473-3099(11)70006-8 21616456 10.1016/S1473-3099(11)70006-8

[62] Silva PE, Costa PS, Ávila MP, Suhadolnik MLS, Reis MP, Salgado APC, et al Leprous lesion presents enrichment of opportunistic pathogenic bacteria. Springerplus. 2015;4: 187 doi: 10.1186/s40064-015-0955-1 25918684 10.1186/s40064-015-0955-1 PMC4405507

