# Supplementary figures and images for: Bacterial diversity in Buruli ulcer skin lesions: Challenges in the clinical microbiome analysis of a skin disease
Source: PLoS One. 2017 Jul 27;12(7):e0181994. doi: 10.1371/journal.pone.0181994 (PMC5531519; doi:10.1371/journal.pone.0181994)

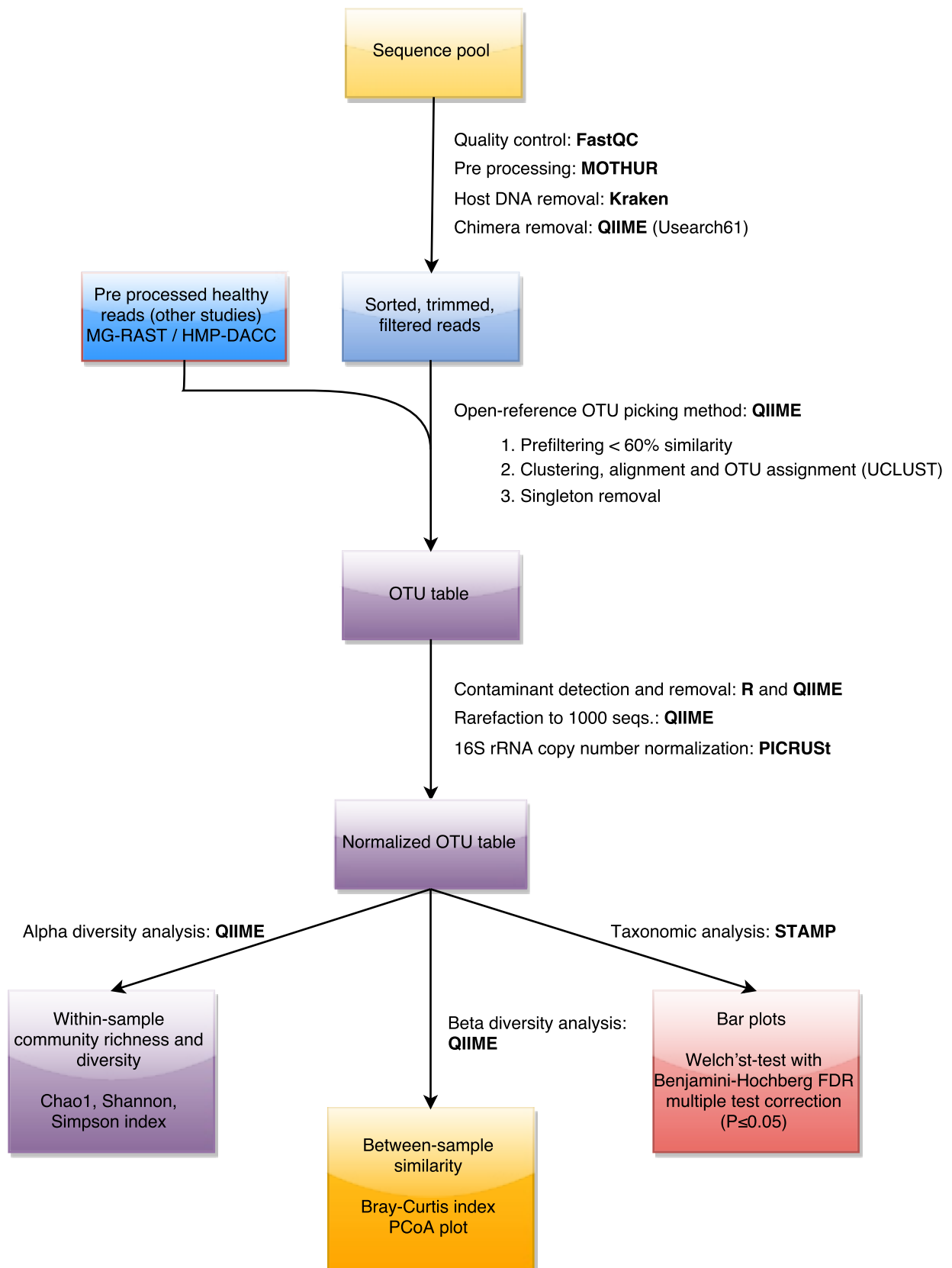

Supplement: S1 Fig — (PDF) [file pone.0181994.s001.pdf]

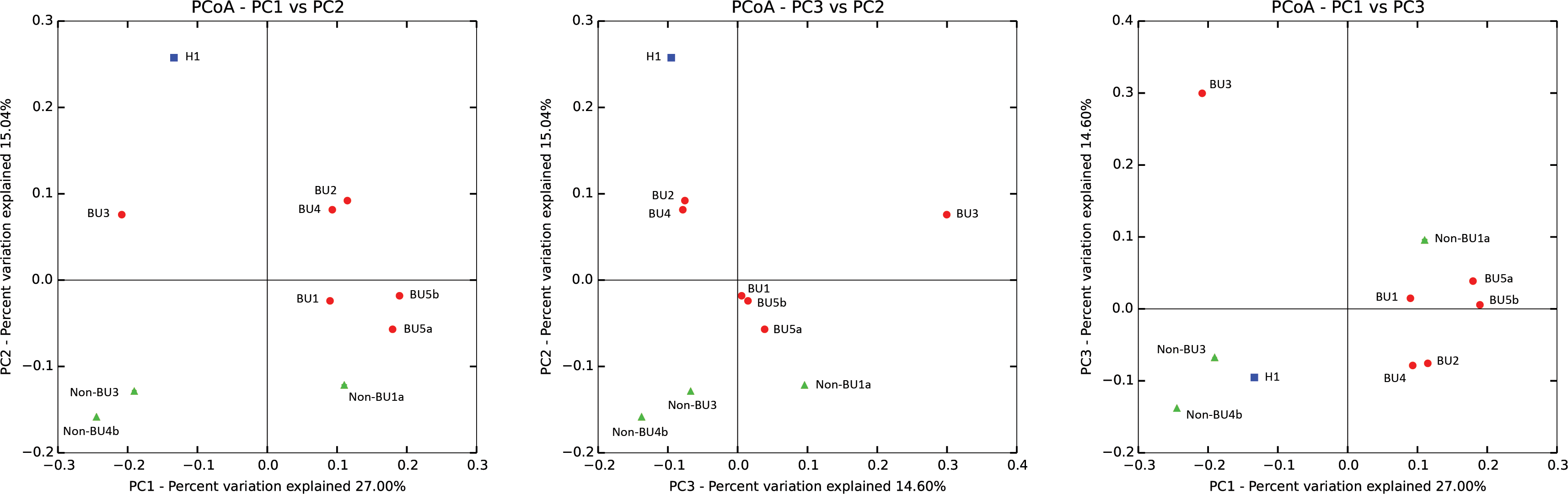

Supplement: S2 Fig — Each point represents a different sample, while the colored circles, triangles, and squares represent BU, non-BU, and Healthy, respectively. In this plot we did not integrate the two samples from the healthy US citizens. (PNG) [file pone.0181994.s002.png]

## Genus classification

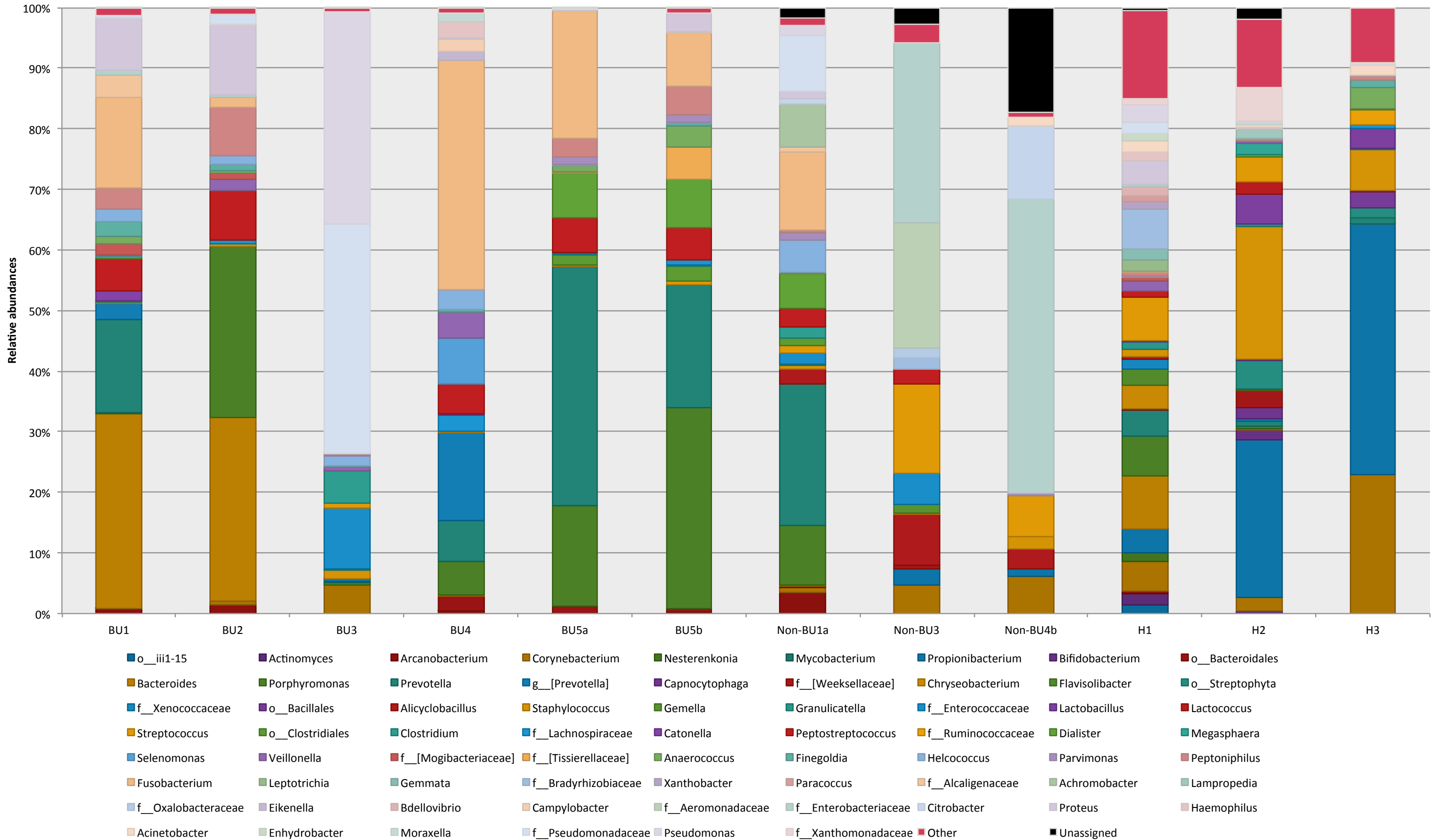

Supplement: S3 Fig — (PDF) [file pone.0181994.s003.pdf]

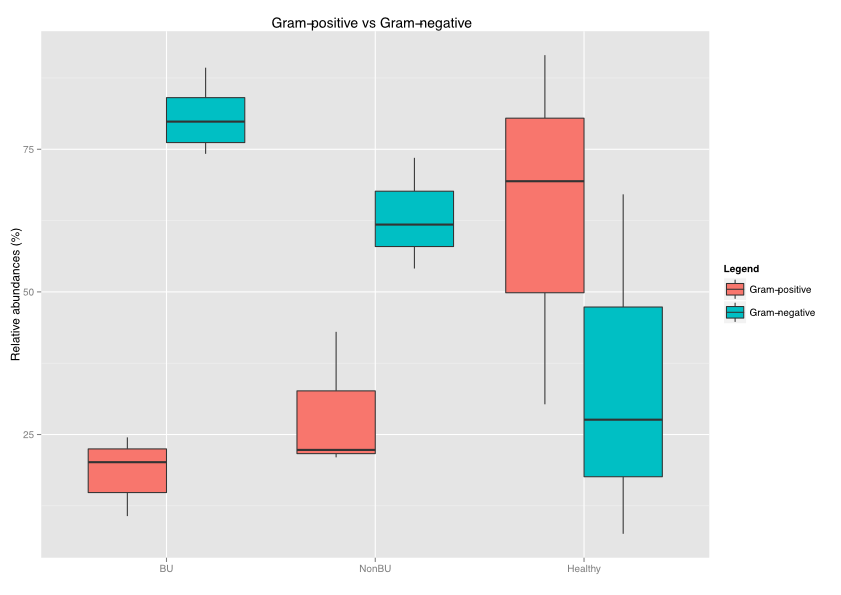

Supplement: S4 Fig — Whiskers in the boxplot represent minimum and maximum values within the three groups. The results show an increase in Gram-negative bacteria within the diseased groups compared to the healthy individuals, although this is not significant. (TIFF) [file pone.0181994.s004.tiff]
