# Supplementary material for: Bacterial diversity in Buruli ulcer skin lesions: Challenges in the clinical microbiome analysis of a skin disease
Source: PLoS One. 2017 Jul 27;12(7):e0181994. doi: 10.1371/journal.pone.0181994 (PMC5531519; doi:10.1371/journal.pone.0181994)
Supplement: S1 Table — This table contains the OTUs and the genera and families (*) they represent. These OTUs were considered contaminants due to a significant inverse spearman correlation and were removed from the dataset. (DOCX) [file pone.0181994.s005.docx]

| **Taxonomic classification** | **OTU identifier** |
| --- | --- |
| *Dietzia* | 553623 |
| *Flavobacterium* | 1145553 |
| *Pedobacter* | 106242, 163090, 1088120, 317024, New.CleanUp.ReferenceOTU42378 |
| Caulobacteraceae* | 285497, 288283, 310003 |
| Bradyrhizobiaceae* | 1105814 |
| *Methylobacterium* | 4323871, 542475, 574655 |
| *Agrobacterium* | 696234 |
| *Paracoccus* | 806547, 590586 |
| *Sphingomonas* | 992510, 582921, 965129 |
| Alcaligenaceae* | 160609, 516182 |
| Comamonadaceae* | 576785, 720353, 3926677 |
| *Comamonas* | 558170, New.CleanUp.ReferenceOTU44590 |
| *Delftia* | 525199, New.CleanUp.ReferenceOTU44308 |
| Enterobacteriaceae* | 581021 |
| *Acinetobacter* | 515265, 873972, 848267, 521318, 706432, 209511, 706432, 209511, 4425634, 521318, 522240, 512485, 265496, 219826, 64653, 532569, 573124, New.ReferenceOTU13, New.CleanUp.ReferenceOTU29162, New.CleanUp.ReferenceOTU826, New.ReferenceOTU71, New.CleanUp.ReferenceOTU29162 |
| *Pseudomonas* | New.CleanUp.ReferenceOTU9452 |
| Unassigned | New.CleanUp.ReferenceOTU35682, New.CleanUp.ReferenceOTU25811, New.CleanUp.ReferenceOTU43488, New.CleanUp.ReferenceOTU37410, New.CleanUp.ReferenceOTU38145, New.CleanUp.ReferenceOTU50506, New.CleanUp.ReferenceOTU9403, New.CleanUp.ReferenceOTU23223 |
